# An Anatomical Study of Maxillary-Zygomatic Complex Using Three-Dimensional Computerized Tomography-Based Zygomatic Implantation

**DOI:** 10.1155/2017/8027307

**Published:** 2017-12-11

**Authors:** Xiangliang Xu, Shijie Zhao, Hui Liu, Zhipeng Sun, Jianwei Wang, Weiguang Zhang

**Affiliations:** ^1^Department of Oral and Maxillofacial Surgery, Peking University School and Hospital of Stomatology, Beijing 100081, China; ^2^Department of Radiology, Peking University Shougang Hospital, Beijing 100041, China; ^3^Department of Medical Radiology, Peking University School and Hospital of Stomatology, Beijing 100081, China; ^4^Department of Anatomy and Histology and Embryology, Peking University Health Science Center, Beijing 100191, China

## Abstract

**Objective:**

To obtain anatomical data of maxillary-zygomatic complex based on simulating the zygomatic implantation using cadaver heads and three-dimensional computerized tomography (3D-CT).

**Methods:**

Simulating zygomatic implantation was performed using seven cadaver heads and 3D-CT images from forty-eight adults. After measuring the maxillary-zygomatic complex, we analyzed the position between the implantation path and the maxillary sinus cavity as well as the distance between the implantation path and the zygomatic nerve.

**Results:**

The distance from the starting point to the endpoint of the implant was 56.85 ± 5.35 mm in cadaver heads and 58.15 ± 7.37 mm in 3D-CT images. For the most common implantation path (80.20%), the implant went through the maxillary sinus cavity completely. The projecting points of the implant axis (IA) on the surface of zygoma were mainly located in the region of frontal process of zygomatic bone close to the lateral orbital wall. The distances between IA and zygomatic nerve in 53 sides were shorter than 2 mm.

**Conclusion:**

The simulating zygomatic implantation on cadaver skulls and 3D-CT imaging provided useful anatomical data of the maxillary-zygomatic complex. It is necessary to take care to avoid the zygomatic nerve injury during implantation, because it frequently appears on the route of implantation.

## 1. Introduction

Endosseous implantation is a standard procedure for edentulous patients [1]. Lacking of sufficient maxilla bone volume, due to bone resorption or pneumatization of the sinus, usually leads to complications and difficulties during implantation [2]. Therefore, implantation in the posterior maxilla region requires augmentation of the bone volume and repeated autologous bone grafting [3]. This procedure significantly increases the successful rate of bone augmentation and implantation; however, the extra surgery for bone harvesting under general anesthesia may increase infection changes and is cost-ineffective [3, 4]. Complications due to bone grafting include sinusitis and loss of grafts [5]. Established by Brånemark in 1997, zygomatic implantation became an innovative method of endosseous implantation in the posterior maxilla region [6, 7]. The implant is usually inserted from the crest of the alveolar process, along the lateral wall of maxillary sinus or through the cavity of maxillary sinus, and ends inside the zygoma bone [8]. Zygomatic implantation has a significant higher success rate, avoiding the disadvantages of bone grafting [9–11]. Due to the extra length of the implant body and the complicated structure of maxillary-zygomatic complex, it is essential to decide the insertion route. Deviations on the inserting angle may lead to severe damage including lateral orbit wall penetration that might hurt the eye ball or perforating zygoma bone into infratemporal fossa. The structures of zygoma bone exhibit individual variations, which makes the anatomical study challenging. To date, researchers are working on collecting anatomical information of zygomatic implantation, the majority obtained from cadavers or zygomatic bone specimens, using either histomorphometry or manual measurement [1, 8, 12]. In 2003, Van Steenberghe et al. simulated the installation of the implants in human cadavers, using an adopted 3D-CT planning system with surgical drilling guide. However, they did not measure the maxilla or the distance between the implants from zygomatic nerves [13]. Although the reported successful rate of zygomatic implantation was 95.8%–100% [10], most of the clinic research did not include randomized controlled trials (RCTs) to confirm efficacy of zygomatic implants in comparison with traditional bone augmentation procedures. Navigation technique has been used in zygomatic implantation recently [14]. It is a valuable tool, and it would play a more important role in the surgical procedure if carried out with more detailed anatomic information of maxillary-zygomatic complex.

In the current study, we performed CT scanning and 3D reconstruction, mimicked the inserting routes of implants using 3D-CT head images, and analyzed the anatomical structures during zygomatic implantation. Our results provided detailed anatomical information for future zygomatic implantation procedures.

## 2. Materials and Methods

### 2.1. Subjects

**Figure 1:**
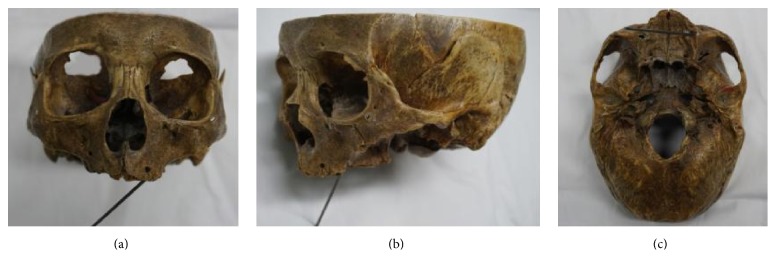
The implantation path on dry skull. (a) Anterior view; (b) lateral view; (c) cranial base view.

**Figure 2:**
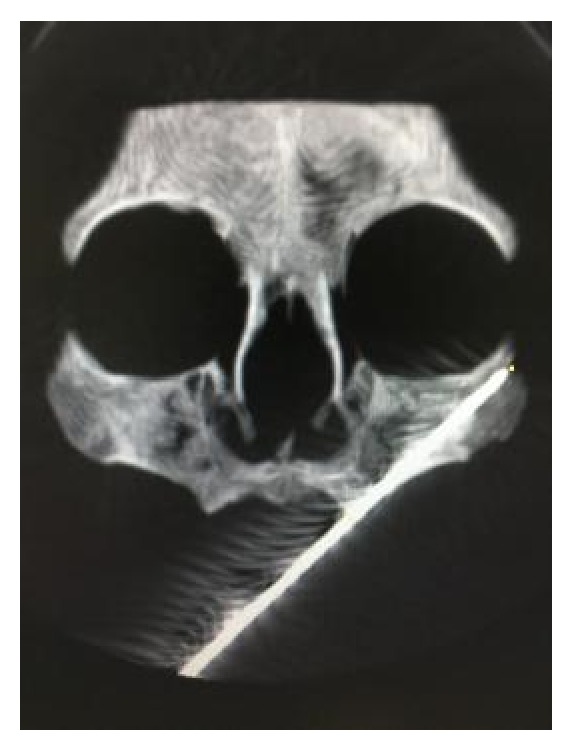
The implantation path on dry skull under CT scanning.

**Figure 3:**
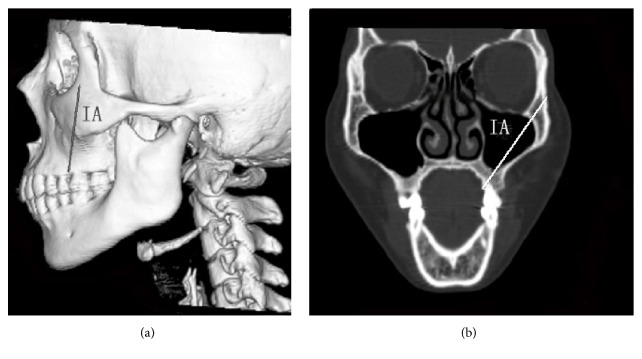
The implant axis (IA) on a 3D-CT image. (a) 3D-CT reconstruction image of the head; (b) CT image of the plane including IA.

We collected seven cadaver heads with edentulous jaws (ages from 60 to 80, with the average 75) from the Department of Anatomy and Histology and Embryology in Peking University Health Science Center to perform simulation of zygomatic implantation (Figures 1 and 2). 3D-CT head images of 48 patients (counting both sides, *n* = 96) without zygomatic, maxillary, or sinus diseases were obtained from the Department of Medical Radiology in Peking University School and Hospital of Stomatology (Figure 3). 27 males and 21 females were enrolled in the study, including 7 patients with edentulous jaws. The ages of the patients ranged from 17 to 89, with the average of 45.

### 2.2. The Simulation of Zygomatic Implantation on the Skulls and CT Scanning

In order to confirm the reliable implantation route, we performed zygomatic implantation simulation using cadaver heads with edentulous jaws with Strong Series Micro Motor Handpiece (STRONG 208-102 WL) (Saeshin Precision Co., Ltd., Daegu, Korea) and long dental drills (Figure 1). Brånemark method [6] was used during the implant installation. An imaginary line was drawn vertically to the infraorbital foramen intersecting with the top of alveolar crest. The starting point of the implant was 5 mm to the palatal side from the intersection point. The clinicians need to be cautious to avoid the implantation route going through the orbit wall or the infratemporal fossa. The implantation route terminated at the surface of the zygoma. We evaluated the following after the procedure: the width of the alveolar crest at the starting point, the location of the endpoint at the zygoma surface, and the completeness of the lateral orbit wall and the front wall of infratemporal fossa.

Then spiral CT scanning images (light speed plus 4, General Electric, Milwaukee, WI, USA) of the cadaver heads were collected (1.25 mm thick; 120 kV, 180 mA) for further evaluation (Figure 2). We measured the angle between the line crossing the bilateral infraorbital foramen (LBIF) and the implantation route (∠*α*), the position between the implantation route and the maxillary sinus, and the distance between the starting and the end points.

### 2.3. The Simulation of Zygomatic Implantation on the CT Scanning Images

**Figure 4:**
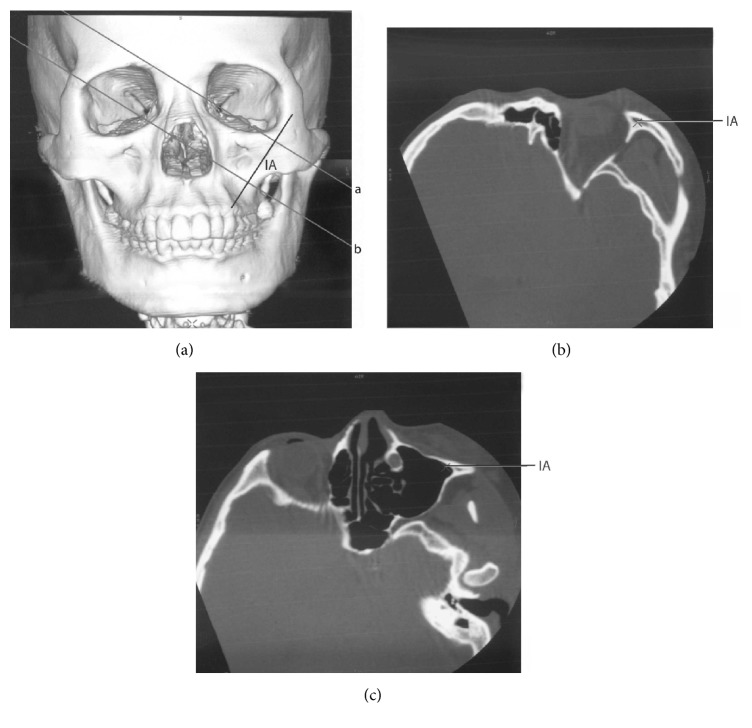
The implant axis (IA) and its surrounding structures. Lines a and b in (a) represented the two planes vertical to IA in the 3D-CT image in (a). Line a went through the bone body of zygoma, while line b went through the maxillary sinus. (a) 3D-CT image of the head. (b) The plane that line a went through, showing the structures around IA in the zygoma. The marked point in the figure indicated IA. (c) The plane that line b went through, showing the position of IA and the maxillary sinus. The marked point in the figure indicated IA.

Spiral CT scanning images (light speed plus 4, General Electric, Milwaukee, WI, USA) from the patient heads were collected (same positions as the cadavers). To simplify the measurement of the anatomical structures of maxilla and zygoma, the indications listed below were followed when simulating the zygomatic implantation on the 3D-CT images (implantation diameter: 4 mm) based on the standard of implantation [8, 15–17] (Figures 3 and 4).

(a) For the patients with dentulous jaws, the insertion location of the installation was at the palatal top of the alveolar crest between the first and the second molars; for the patients with edentulous jaws, the insertion location of the installation was 5 mm to the palatal side from the point of the intersection of the vertical line going through the infraorbital foramen and the top of alveolar crest.

(b) To obtain the maximum support from the bone body, the implantation route went through bony structures as deeply as possible, including maxillary alveolar process, bone wall of maxillary sinus, and zygoma bone body. The thickness of the zygomatic bone surrounding the implantation route should be no less than 1 mm.

(c) To avoid injuries, the implantation route should not go through the orbit wall to avoid eye ball injuries, or go through the infratemporal fossa to avoid interfering infratemporal space.

**Figure 5:**
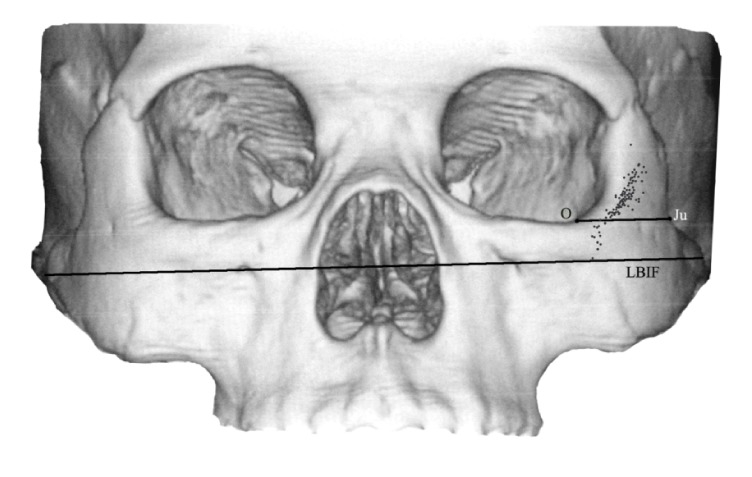
The projecting points of IA extension on the surface of the zygoma and the width of the zygoma. The jugale (Ju) point was the most depressed point on the lateral surface of the zygoma. LBIF referred to the line crossing the bilateral infraorbital foramina. The point O was defined as the point where the line through point Ju parallel to LBIF crossed the lateral margin of the orbit.

**Figure 6:**
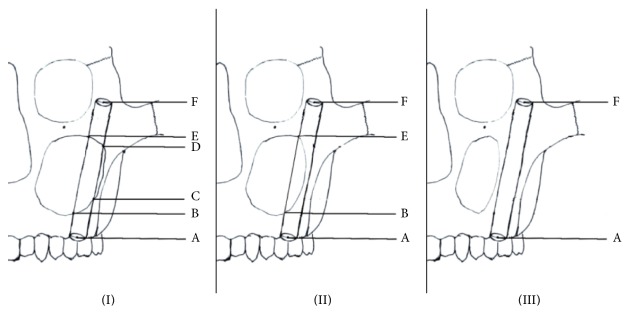
Three types (types (I), (II), and (III)) of position relationships between the implant and the maxillary sinus cavity. The starting point of IA on the alveolar crest was defined as point A. If the implant entered the sinus cavity, the point at which it first entered the sinus cavity was defined as point B. The point at which the implant was entirely inside the sinus was defined as point C, and the point at which the implant began to reenter bone was defined as point D. The point at which the implant exited the sinus wall and completely entered bone was defined as point E. The endpoint of IA was defined as point F.

With GE advantage workstation software version 4.0 (General Electric, Milwaukee, WI, USA), the 3D-CT images with implant axis (IA) were rotated to obtain the tomographic images vertical to IA. These images indicated the structure around the implant clearly, including the position of the implant and the bony structure, the thickness of the bones surrounding the implant, where the implant entered the maxillary sinus and the zygomatic bone body, and the location of the zygomatic nerve. Measurements of the following parameters were performed based on the 3D-CT images: the width and the thickness of zygoma (Figure 5), the width of the alveolar crest of the implant starting point, the angle between the line crossing the bilateral infraorbital foramen (LBIF) and IA (∠*α*), the distribution of the crossing points of IA extension line and the zygomatic surface (Figure 5), the position of the zygomatic implant and the maxillary sinus (Figure 6), the position of the zygomatic implant and the zygomatic nerve, and the distance between the starting point and the endpoint of the zygomatic implant (Figure 6).

In Figure 5, the projecting points of IA extension on the surface of the zygoma and the width of the zygoma were shown. The jugale (Ju) point was the most depressed point on the lateral surface of the zygoma, where the lateral margin of the zygomaticofrontal process met the upper margin of the zygomaticotemporal process. Point O was defined as the point where the line through point Ju parallel to the line crossing the bilateral infraorbital foramina (LBIF) crossed the lateral margin of the orbit. The distance between point Ju and point O was defined as the width of the zygoma. The distance between the internal surface and the external surface of zygoma at the midpoint of the line Ju-O was defined as the thickness of the zygoma. The spots on the surface of left zygoma show the relative positions of the projecting points of IAs determined in all cases.

In Figure 6, three types of position relationships between the implant and the maxillary sinus cavity were shown: type I (part of the implant was completely inside the sinus cavity and unsupported by the bone body); type II (part of the implant entered the sinus cavity and part remained embedded in the sinus bone wall); type III (the middle part of the implant is totally located inside of the maxillary sinus bone walls and no part of the implant entered the sinus cavity). The points A, B, C, D, E, and F could be seen in Figure 6. We measured the distances between the two proximate points (AB, BC, CD, DE, and EF) and calculated the distance between the starting point and the endpoint of IA, the lengths of the part partially in the sinus cavity (BC + DE), and the part totally embedded in bone (AB + EF).

### 2.4. Statistical Analysis

One clinician performed all the measurements to minimize error. The data were presented as mean ± standard deviation. Unpaired *t*-test was applied. *P* values equal to or smaller than 0.05 were considered significant. All analysis was performed using SPSS version 11.5 (SPSS Inc., Chicago, IL, USA).

## 3. Results

### 3.1. The Simulating Zygomatic Implantation on the Skulls and the CT Scanning

We found that the width of the alveolar crest at the implant starting point was 3 ± 0.52 mm using the seven skull specimens. All the endpoints of the implants were located on the surface of the zygoma near the orbit wall. All specimens had intact lateral orbit walls and front walls of infratemporal fossa after the installation.

The CT scanning showed the following results: the angle between LBIF and the implantation route (∠*α*) was 56.54 ± 2.05°; all the implant routes went through the maxillary sinus; the distance between the starting point and the endpoint was 56.85 ± 5.35 mm.

### 3.2. The Simulation of Zygomatic Implantation on the CT Scanning Images

#### 3.2.1. The Width of the Alveolar Crest at the Implant Starting Point

The width of the alveolar crest at the implant starting point in male patients (11.62 ± 1.47 mm) was significantly longer than the width in female patients (10.78 ± 2.02 mm) (*P* < 0.05, Table 1).

#### 3.2.2. The Distance between the Starting Point and the Endpoint and the Angle between the Line Crossing the Bilateral Infraorbital Foramen (LBIF) and IA (∠*α*)

Overall, the distance (mean ± SD) between the starting point and the endpoint of the implant in all the patients was 58.15 ± 7.37 mm (ranging from 36.46 to 72.35 mm). The distance in male patients (59.59 ± 7.50 mm) was significantly longer than the distance in female patients (56.29 ± 6.85 mm) (*P* < 0.05). The implants had various distances in different tissues/bone bodies (Table 2). The portion inside the bone body was longer than 10 mm in the majority of the implants, except for 2 cases (5.94 mm and 8.70 mm, 2.08% of the total cases). The angle between the line crossing LBIF and IA (∠*α*) in male patients (55.93 ± 3.48°) was significantly greater than the angle in female patients (53.73 ± 4.89°) (*P* < 0.05).

#### 3.2.3. The Distribution of the Crossing Points of IA Extension Line and the Zygomatic Surface

The crossing points were distributed on the zygomatic surface near the lateral orbit wall. To simplify the analysis of the distribution, we set an imaginary line through the jugale point (Ju) that was parallel to LBIF (Ju-O line, Figure 3). Our results indicated that 87.5% of the crossing points of IA extension line and the zygomatic surface fell above or on the Ju-O lines. The crossing points that fell above the Ju-O lines had a distance of 5.64 ± 3.34 mm from the Ju-O lines, whereas the ones that fell below the Ju-O lines had a distance of 3.17 ± 2.00 mm from the Ju-O lines.

#### 3.2.4. The Positions of the Zygomatic Implant and the Maxillary Sinus

We summarized three categories of positions of the zygomatic implant and the maxillary sinus (Figure 5 and Table 2).

(I) The medial part of the implant was completely inside the sinus cavity and not supported by the bone body (80.20%).

(II) Part of the implant entered the sinus cavity and part remained embedded in the sinus bone wall (15.63%).

(III) The medial part of the implant was totally located inside of the maxillary sinus bone walls and did not enter the sinus cavity (4.17%).

#### 3.2.5. The Position of the Zygomatic Implant and the Zygomatic Nerve

The zygomatic nerves were found in all patients using CT images. 53 cases (sides) (55.21% of all cases) had the shortest distance between IA and zygomatic nerve less than 2 mm.

#### 3.2.6. The Comparisons between the Patients with Dentulous Jaws and with Edentulous Jaws

On average, the patients with edentulous jaws were 32.19 years older than the ones with dentulous jaws (*P* < 0.05). The angles between the line crossing the bilateral infraorbital foramen (LBIF) and IA (∠*α*) in the patients with edentulous jaws were 3.27° smaller than the ones in the patients with dentulous jaws (*P* < 0.05), whereas the widths of alveolar crest at the starting point were 1.28 mm narrower compared to the patients with dentulous jaws (*P* < 0.05). The other measurements did not exhibit significant difference between the two types (Table 3).

## 4. Discussion

In the present study, we simulated the zygomatic implantation on cadaver skulls and 3D-CT head images and measured the anatomical parameters of the area around the implantation route, including the length of the implant inside the bone body, the distance between the implant and the zygomatic nerve, and the position of the implant in/around the maxillary sinus bone wall. Most of the previous studies on maxilla and zygoma that are related to the application of zygomatic implantation were done with traditional methods, including direct measurements on cadaver samples and tissue morphological analysis [1, 8, 12]. However, these traditional methods were not perfect. For instance, during direct measurements, the starting points and endpoints of the zygomatic implantation were all at the same point without considering the individual anatomical variations [8]. The surfaces of the zygomatic-maxillary complex have relatively large curvatures that vary in different individuals when working with patients. The combination of all information determines the range of the simulating implant route that the clinicians should follow to avoid injuries, including penetrations into maxillary sinus front wall, lateral orbit wall, or infratemporal fossa. In addition, traditional methods were not suitable for intact samples, because it was difficult to obtain the structural details about the surrounding tissues. In 2003, Nkenke et al. performed morphological analysis of the implant surrounding tissues, through sawing the region of the second premolar perpendicular to the zygomaticoalveolar ridge in the plane of the intended direction of implant installation [1]. Apparently, finding the intended direction and plane of implant placement is difficult without advances in imaging. In the current study, we simulated the zygomatic implantation with 3D-CT images before the measurements which allowed us to find an ideal direction and plane for implantation.

Choosing a proper technique for the implant installation is essential for the entire procedure. In 2013, Chrcanovic et al. compared five different zygomatic implantation methods and pointed out the advantages as well as the complications such as the anatomical structures of alveolar crest, maxillary sinus, and zygoma [18]. To facilitate our procedure, we chose the classical Brånemark method for the simulating zygomatic implantation. Ideally, the implant would be located inside the zygomatic-maxillary complex without perforating zygoma or being exposed towards mucous membrane, which eliminated the risk of infection.

There are two important aspects in zygomatic implantation: the zygomatic-maxillary complex and the implant. With more information and data that we could obtain, we will achieve better outcomes during the procedure. Many studies have focused on the linear and angular measurements for the insertion of zygomatic implants [8, 19, 20]. In 2011, Corvello et al. evaluated the length of the holes drilled during the zygomatic implantation and compared the two surgical methods including the original Brånemark and the Exteriorized protocols [20]. They found that the Exteriorized technique increased the length of the drilling holes which might provide more support for zygomatic implants. In addition, among the 13 sections on the zygomatic bone surface, the most frequent positions where the implants emerged were sections 9 and 12 in both methods. We confirmed this result. Takamaru et al. demonstrated the importance of obtaining the height and the thickness of the zygomatic bone [21]. They designated eight points as landmarks and the thickness of each part of the zygomatic bone was measured using calipers. The thickness of the zygomatic bone at the 90° angle point where the apex of the implant penetrates was 1.8 ± 0.4 mm, which was thinner than the diameter of the implant (2.8 mm), indicating that another insertion method is required. However, these studies were performed on the surface of dry skulls and they did not consider the anatomical structure of the zygomatic bone.

Here, we used the 3D-CT images in the planes vertical to the implant axis instead of other planes (sagittal, coronal, and horizontal), to provide the anatomical data of the zygomatic-maxillary complex closer to the zygomatic implant. Based on the position of the implant route to the maxillary sinus, we divided the cases into three categories (as described in Results). 80.20% of the cases were type I that the middle part of the implant went through the maxillary sinus cavity completely. However, the average length of the implant embedded inside the bone was 26.10 mm in type I patients, indicating that most of the zygomatic implants had enough support from maxilla and zygoma. We found that two cases in type I patients had embedded part of the implants less than 10 mm, which could not provide enough support from the bone body when installing zygomatic implantation. Type III patients had smaller maxillary sinuses and thicker bone walls, which could provide enough bone body to support. For type III patients, clinicians could choose shorter implants in order to simplify the procedure and eliminate the risk of complications. According to our results, most of the patients in the present study had zygomatic-maxillary complexes that were suitable for the zygomatic implantation. The distance between the implant starting point and the endpoint was 58.15 ± 7.37 mm, which could be used as a reference in future clinical practice.

Another concern of zygomatic implantation is zygomatic nerve (nervus zygomaticofacialis) injuries. In 2003, Nkenke et al. performed histological analysis in the plane of the intended direction of implant installation. In 19 out of 30 specimens the nervus zygomaticofacialis was encountered. They also predicted that the zygomatic nerve injuries may occur during the implantation [1]. We detected the zygomatic nerves in the zygomas of all patients with the help of 3D-CT scanning. 55.21% of the patients had a shortest distance between the zygomatic nerve and the implant route for less than 2 mm. The diameter of the implants was 4 mm. It is possible that the implant installation would cause zygomatic nerve injuries. Since the zygomatic nerve projects to the skin around the zygomaticotemporal area, sensitivity disorder of the malar skin might happen. Some authors have reviewed the complications of zygomatic implantation [22, 23]. The damage of zygomaticofacial nerves could occur during clinical procedure of implant placement. According to our study, proper preoperative design and careful preoperative examination, especially CT images, should be performed to check the position of zygomatic nerve and avoid injuring it. Consideration of zygomatic nerve before case planning would be very important particularly for those surgeons who just begin to use the zygomatic implants.

We discovered that the crossing points of IA extension line and the zygomatic surface are mostly distributed on the surface of frontal process of zygomatic bone near the lateral orbit wall, which was consistent with previous observations [7]. We believe that the smaller angles between LBIF and IA (∠*α*) in the patients with edentulous jaws compared to dentulous jaws contribute to the resorption of alveolar bones.

## 5. Conclusion

We performed the simulation of zygomatic implantation in 7 cadaver skulls and 3D-CT images from 48 patients (96 sides), measured the parameters involved in the implant installation, and obtained detailed and reliable anatomical data of the zygomatic-maxillary complex. The application of 3D-CT scanning could be utilized as a resourceful guidance during the zygomatic implantation in the future to choose the optimal routes and protect the zygomatic nerve.

## Figures and Tables

**Table 1:** Measurements in male and female patients.

	Gender		*P* value
	Male (mean ± SD)	Female (mean ± SD)	
Age	43.59 ± 19.62	46.33 ± 22.61	0.527
The width of zygoma (mm)	20.72 ± 2.30	19.66 ± 2.94	0.051
The thickness of zygoma (mm)	5.67 ± 1.16	5.62 ± 1.49	0.838
The distance between the starting point and endpoint (mm)	59.59 ± 7.50	56.29 ± 6.85	0.029
The width of the alveolar crest at the starting point (mm)	11.62 ± 1.47	10.78 ± 2.02	0.021
∠*α* (°)	55.93 ± 3.48	53.73 ± 4.89	0.015

**Table 2:** The position relationships between the implant and the maxillary sinus cavity.

Type	Sides/percentage	The length of the implant embedded inside the bone body completely (mean ± SD) (mm)	The length of the implant partly inside the sinus cavity (mean ± SD) (mm)	The length of the implant completely inside the sinus cavity (mean ± SD) (mm)
I	77/80.20%	26.10 ± 8.80 (AB + EF)	8.11 ± 5.85 (BC + DE)	24.17 ± 11.06 (CD)
II	15/15.63%	42.47 ± 14.31 (AB + EF)	15.55 ± 7.62 (BE)	/
III	4/4.17%	54.16 ± 4.53 (AF)	/	/

**Table 3:** Measurements in patients with dentulous jaws and with edentulous jaws.

	Patients with dentulous jaws (mean ± SD)	Patients with edentulous jaws (mean ± SD)	*P* value
Age	40.10 ± 18.49	72.29 ± 10.55	0.000
The width of zygoma (mm)	19.46 ± 2.75	20.40 ± 2.61	0.224
The thickness of zygoma (mm)	5.93 ± 1.49	5.60 ± 1.28	0.387
The distance between the starting point and the endpoint (mm)	55.65 ± 6.54	58.57 ± 7.46	0.171
The width of alveolar crest (mm)	9.39 ± 1.97	11.56 ± 1.54	0.000
∠*α* (°)	52.17 ± 5.52	55.44 ± 3.87	0.008

## References

[1] Nkenke E., Hahn M., Lell M. (2003). Anatomic site evaluation of the zygomatic bone for dental implant placement. *Clinical Oral Implants Research*.

[2] Malevez C., Daelemans P., Adriaenssens P., Durdu F. (2003). Use of zygomatic implants to deal with resorbed posterior maxillae. *Periodontology 2000*.

[3] Ten Bruggenkate C. M., Van Den Bergh J. P. A. (1998). Maxillary sinus floor elevation: A valuable pre-prosthetic procedure. *Periodontology 2000*.

[4] Raghoebar G. M., Vissink A., Reintsema H., Batenburg R. H. K. (1997). Bone grafting of the floor of the maxillary sinus for the placement of endosseous implants. *British Journal of Oral and Maxillofacial Surgery*.

[5] Kahnberg K.-E., Ekestubbe A., Gröndahl K., Nilsson P., Hirsch J.-M. (2001). Sinus lifting procedure. I. One-stage surgery with bone transplant and implants. *Clinical Oral Implants Research*.

[6] Parel S. M., Brånemark P.-I., Ohrnell L.-O., Svensson B. (2001). Remote implant anchorage for the rehabilitation of maxillary defects. *Journal of Prosthetic Dentistry*.

[7] Brånemark P. I., Adell R., Albrektsson T., Lekholm U., Lindström J., Rockler B. (1984). An experimental and clinical study of osseointegrated implants penetrating the nasal cavity and maxillary sinus. *Journal of Oral and Maxillofacial Surgery*.

[8] Uchida Y., Goto M., Katsuki T., Akiyoshi T. (2001). Measurement of the maxilla and zygoma as an aid in installing zygomatic implants. *Journal of Oral and Maxillofacial Surgery*.

[9] Malevez C., Abarca M., Durdu F., Daelemans P. (2004). Clinical outcome of 103 consecutive zygomatic implants: A 6–48 months follow-up study. *Clinical Oral Implants Research*.

[10] Esposito M., Worthington H. V. (2013). Interventions for replacing missing teeth: dental implants in zygomatic bone for the rehabilitation of the severely deficient edentulous maxilla.. *Cochrane Database of Systematic Reviews*.

[11] Zwahlen R. A., Grätz K. W., Oechslin C. K., Studer S. P. (2006). Survival rate of zygomatic implants in atrophic or partially resected maxillae prior to functional loading: A retrospective clinical report. *The International Journal of Oral & Maxillofacial Implants*.

[12] Rigolizzo M. B., Camilli J. A., Francischone C. E., Padovani C. R., Brånemark P.-I. (2005). Zygomatic bone: Anatomic bases for osseointegrated implant anchorage. *The International Journal of Oral & Maxillofacial Implants*.

[13] Van Steenberghe D., Malevez C., Van Cleynenbreugel J. (2003). Accuracy of drilling guides for transfer from three-dimensional CT-based planning to placement of zygoma implants in human cadavers. *Clinical Oral Implants Research*.

[14] Gasparini G., Boniello R., Laforì A. (2017). Navigation system approach in zygomatic implant technique. *The Journal of Craniofacial Surgery*.

[15] Vrielinck L., Politis C., Schepers S., Pauwels M., Naert I. (2003). Image-based planning and clinical validation of zygoma and pterygoid implant placement in patients with severe bone atrophy using customized drill guides. Preliminary results from a prospective clinical follow-up study. *International Journal of Oral and Maxillofacial Surgery*.

[16] Boyes-Varley J. G., Howes D. G., Lownie J. F., Blackbeard G. A. (2003). Surgical modifications to the Branemark zygomaticus protocol in the treatment of the severely resorbed maxilla: a clinical report. *The International Journal of Oral & Maxillofacial Implants*.

[17] Chow J., Hui E., Lee P. K. M., Li W. (2006). Zygomatic Implants-Protocol for Immediate Occlusal Loading: A Preliminary Report. *Journal of Oral and Maxillofacial Surgery*.

[18] Chrcanovic B. R., Pedrosa A. R., Custódio A. L. N. (2013). Zygomatic implants: A critical review of the surgical techniques. *Journal of Oral and Maxillofacial Surgery*.

[19] Rossi M., Duarte L. R., Mendonça R., Fernandes A. (2008). Anatomical bases for the insertion of zygomatic implants. *Clinical Implant Dentistry and Related Research*.

[20] Corvello P. C., Montagner A., Batista F. C., Smidt R., Shinkai R. S. (2011). Length of the drilling holes of zygomatic implants inserted with the standard technique or a revised method: A comparative study in dry skulls. *Journal of Cranio-Maxillo-Facial Surgery*.

[21] Takamaru N., Nagai H., Ohe G. (2016). Measurement of the zygomatic bone and pilot hole technique for safer insertion of zygomaticus implants. *International Journal of Oral and Maxillofacial Surgery*.

[22] Molinero-Mourelle P., Baca-Gonzalez L., Gao B., Saez-Alcaide L.-M., Helm A., Lopez-Quiles J. (2016). Surgical complications in zygomatic implants: A systematic review. *Medicina Oral Patología Oral y Cirugía Bucal*.

[23] Chrcanovic B. R., Albrektsson T., Wennerberg A. (2016). Survival and complications of zygomatic implants: an updated systematic review. *Journal of Oral and Maxillofacial Surgery*.

